# EGCG Derivatives Alleviate Diquat-Induced Liver and Gut Damage in Mice by Activating an Antioxidant Pathway and Enhancing Barrier Function

**DOI:** 10.3390/ani16060966

**Published:** 2026-03-19

**Authors:** Liting Xu, Caiwei Luo, Xuyang Gao, Jianmin Yuan, Bin Fu

**Affiliations:** 1 College of Science, China Agricultural University, Beijing 100193, China; 2 State Key Laboratory of Animal Nutrition and Feeding, College of Animal Science and Technology, China Agricultural University, Beijing 100193, China

**Keywords:** EGCG derivatives, oxidative stress, intestinal barrier function, antioxidant capacity, Nrf2/HO-1 signaling pathway

## Abstract

(−)-Epigallocatechin 3-gallate (EGCG) is a potent natural antioxidant, but its strong bitterness and poor palatability limit its application in animal production. This study aimed to evaluate the protective effects and underlying mechanisms of chemically synthesized EGCG derivatives against oxidative stress using a diquat-induced mouse model. A total of 36 male ICR mice were randomly assigned into six groups (n = 6): Control (T0), Diquat (T1), EGCG + Diquat (T2), Epigallocatechin octanoate (EGCO) + Diquat (T3), Epigallocatechin *p*-chloromethylbenzoate (EGCP) + Diquat (T4), and Epigallocatechin ibuprofen ester (EGCI) + Diquat (T5). Oxidative stress was induced by intraperitoneal injection of diquat at day 27 of the experiment, while EGCG or its derivatives were administered via dietary supplementation. At day 28, the mice were weighed, killed, and the tissues were sampled. Diquat challenge significantly impaired growth, increased serum injury markers (ALT, AST, DAO, and D-LA) (*p* < 0.05), suppressed hepatic and jejunal antioxidant enzymes (GPx, SOD, and TAOC) while elevating MDA (*p* < 0.05), damaged jejunal morphology (villus atrophy) (*p* < 0.05), and downregulated tight junction proteins (ZO-1 and Occludin) (*p* < 0.05). Chemically synthesized EGCG derivatives, especially EGCI, effectively alleviated diquat-induced growth impairment and hepatic and intestinal oxidative damage by improving intestinal barrier function and enhancing systemic antioxidant capacity, possibly in part through activation of the nuclear factor erythroid 2-related factor 2/heme oxygenase-1 (Nrf2/HO-1) signaling pathway. Compared with EGCG, EGCI exhibited reduced bitterness and improved palatability, which favored normal feed intake. These findings provide strong theoretical support for the future application of EGCG derivatives, especially EGCI, as functional antioxidant additives in broiler production.

## Simple Summary

To meet the rapidly growing global demand for poultry products, broiler breeding generally pursues rapid growth, high feed conversion rate, and high breast meat yield, which also make it more vulnerable to oxidative stress. Although (−)-epigallocatechin 3-gallate (EGCG) is considered a potential functional antioxidant additive, its strong bitterness and poor palatability limit its practical application. Due to the current limitations in the synthesis yield, this study employed a diquat-induced mouse model to evaluate the protective effects of chemically synthesized EGCG derivatives against oxidative stress and to explore their underlying mechanisms. The results demonstrated that these derivatives, particularly epigallocatechin ibuprofen ester, significantly alleviated growth retardation and mitigated oxidative damage in the liver and intestine. Their protective effects, which include improved intestinal barrier function and enhanced systemic antioxidant capacity, were partially mediated by activating a key antioxidant defense pathway in cells—the nuclear factor erythroid 2-related factor 2/heme oxygenase-1 (Nrf2/HO-1) signaling pathway. Compared with EGCG, these derivatives exhibited reduced bitterness and improved palatability, which helped maintain normal feed intake. This study provides a theoretical foundation for the use of EGCG derivatives as effective, low-bitterness antioxidant feed additives in broiler production.

## 1. Introduction

To meet the rapidly growing global demand for poultry products, modern breeding programs have placed strong emphasis on achieving faster growth rates, improved feed efficiency, and increased breast muscle yield in broiler chickens [1,2]. Although these genetic advancements have substantially enhanced production efficiency, they have also increased the metabolic burden on broilers, rendering them more susceptible to oxidative stress. In commercial production systems, broilers are frequently exposed to various oxidative challenges arising from environmental and nutritional sources, including high stocking density, heat stress, oxidized oils, fungal contamination, and mycotoxins in feed, as well as heavy metal pollutants [3,4,5]. These stressors stimulate excessive production of reactive oxygen species (ROS), ultimately inducing oxidative damage. In broilers, this damage impairs immunity, intestinal barrier function, and microbial homeostasis, which in turn affects growth and meat quality [6,7,8,9], resulting in huge economic losses in global poultry production. Therefore, finding effective nutritional interventions to reduce oxidative stress has become a key focus of contemporary animal nutrition research.

(−)-Epigallocatechin 3-gallate is the most abundant and biologically active catechin polyphenol extracted from green tea. It possesses potent antioxidant, anti-inflammatory, and antimicrobial properties and is widely recognized as a functional feed additive for mitigating oxidative stress [10,11]. Previous studies have reported that EGCG alleviates hepatic oxidative damage in broilers by reducing ROS and malondialdehyde (MDA) accumulation, thereby improving growth performance [12]. Additionally, EGCG enhances intestinal villus morphology and barrier function, strengthening both antioxidant and anti-inflammatory capacities and effectively reducing heat stress induced intestinal oxidative injury [13]. EGCG has also been shown to reverse LPS induced gut microbiota dysbiosis by restoring the *Firmicutes-to-Bacteroidetes* ratio and markedly decreasing the abundance of *Enterobacteriales* [14,15], thereby protecting the host against oxidative stress related damage. Collectively, these findings provide strong evidence that EGCG exerts robust protective effects against oxidative injury induced by various stressors.

However, despite its potent bioactivity, the practical application of EGCG in poultry production remains limited due to its pronounced bitterness, which markedly compromises feed palatability. Supplementation of broiler diets with EGCG significantly reduces feed intake, diminishing its potential growth-promoting effects [16]. In laying hens, the intense bitterness of EGCG has also been associated with reduced feed intake, decreased egg production, and decreased egg quality [17,18]. Previous studies indicate that the bitter taste of EGCG primarily arises from the galloyl group in its molecular structure [19]. To overcome this limitation, our laboratory employed a chemical synthesis approach in which (−)-epigallocatechin (EGC), a catechin lacking a galloyl group, was conjugated with other bioactive antioxidant or anti-inflammatory compounds to generate a series of novel EGCG derivatives. These derivatives were designed to reduce bitterness while maintaining or enhancing biological activity.

Diquat is a highly efficient redox-cycling agent that continuously participates in mitochondrial redox reactions, generating large amounts of superoxide anions and other reactive oxygen species-derived metabolites [20]. This process induces pronounced oxidative stress, lipid peroxidation, and inflammatory responses, thereby disrupting the homeostatic balance between oxidative and antioxidative systems. Owing to its well-defined mechanism of action, substantial capacity to induce oxidative injury, and high reproducibility, diquat has been extensively used to establish in vivo oxidative stress models in mice [21], broilers [22,23], laying hens [24], and other animal species.

The nuclear factor erythroid 2-related factor 2/Heme oxygenase-1 (Nrf2/HO-1) signaling pathway is a central regulatory system for maintaining redox homeostasis and protecting organisms against oxidative stress [25,26]. Nrf2 is the main transcription factor governing intracellular antioxidant defense. Under oxidative stress, Nrf2 is activated and translocated into the nucleus, where it forms heterodimers with small Maf proteins (v-maf musculoaponeurotic fibrosarcoma oncogene homolog) and subsequently initiates the transcription of antioxidant response element (ARE)-dependent downstream genes [27], thereby enhancing cellular resistance to oxidative injury.

Heme oxygenase-1 (HO-1) is a key target gene regulated by Nrf2. It can degrade heme into biliverdin, carbon monoxide and free iron, and has significant antioxidant, anti-inflammatory and cytoprotective effects [28]. Accumulating studies demonstrate that EGCG potently stimulates the Nrf2 pathway and enhances HO-1 production, thereby suppressing excessive ROS formation, alleviating oxidative stress, and associated inflammatory damage [29,30]. Therefore, the Nrf2/HO-1 pathway is widely regarded as a fundamental molecular basis for the antioxidant biological effects of EGCG.

Collectively, modern broilers are highly susceptible to oxidative stress, underscoring the need for safe and effective nutritional interventions. Although EGCG possesses powerful antioxidant and anti-inflammatory properties, its use in poultry is limited by its strong bitter taste. In this study, novel EGCG derivatives were synthesized to reduce bitterness while preserving bioactivity. Due to limitations in synthesis yield, large-scale broiler trials are temporarily unfeasible. Therefore, a mouse model with diquat-induced oxidative stress was selected to perform preclinical proof-of-concept validation of their systemic antioxidant efficacy and underlying core molecular mechanisms, focusing on the Nrf2/HO-1 pathway. This work provides lead compounds and key scientific evidence for the subsequent development of specialized feed additives aimed at alleviating oxidative stress in poultry.

## 2. Materials and Methods

### 2.1. Chemicals

Diquat (DQ, 99.5%) was bought from Aladdin Chemical Co. (Shanghai, China). (−)-Epigallocatechin 3-gallate (EGCG, 99%) was bought from Heowns Biochem LLC (Tianjin, China). Epigallocatechin octanoate, epigallocatechin *p*-chloromethylbenzoate, and epigallocatechin ibuprofen ester were previously synthesized in our laboratory.

### 2.2. Animal Experiment Design

All animal experiments were approved by the Institutional Animal Care and Use Committee of China Agricultural University (Approval Number: AW01115202-6-01). Male ICR mice (four weeks old) were provided by SPF Biotechnology Co., Ltd. (Beijing, China) and allowed to acclimate for one week under controlled environmental conditions (22–25 °C; 12 h light/dark cycle). Mice were housed in standard polypropylene cages (290 mm × 178 mm × 160 mm) with autoclaved wood shavings as bedding, and had free access to a standard basal diet and clean drinking water. After the adaptation period, a total of 36 male ICR mice were randomly divided into six groups (n = 6 per group) with no significant difference in initial body weight among the groups: Control (T0), Diquat (T1), EGCG + Diquat (T2), Epigallocatechin octanoate (EGCO) + Diquat (T3), Epigallocatechin *p*-chloromethylbenzoate (EGCP) + Diquat (T4), and Epigallocatechin ibuprofen ester (EGCI) + Diquat (T5). Due to constraints on animal housing capacity, mice within each treatment group were housed in two separate cages, with three mice in each cage.

In the T0 and T1 groups, the mice received only the basal diet. In contrast, those in the intervention groups were fed the basal diet supplemented with 300 mg/kg of the respective test compounds (EGCG, epigallocatechin octanoate, epigallocatechin *p*-chloromethylbenzoate, or epigallocatechin ibuprofen ester). The dietary intervention lasted for four weeks. On day 27, all mice except those in the control group were intraperitoneally injected with diquat at 15 mg/kg body weight (BW) (injection volume: 200 μL per mouse). The control group received an equivalent volume of sterile phosphate-buffered saline (PBS).

### 2.3. Assessment of Growth Performance

BW of mice was recorded at the start of dietary intervention and on day 27, immediately before diquat administration. To evaluate the protective effects of the tested compounds against diquat-induced growth inhibition, body weight was measured 24 h after diquat injection again. At the end of the experiment, feed intake (FI) was recorded to provide a descriptive assessment of overall feed consumption across treatment groups. Due to the limited number of cage replicates (n = 2), statistical comparisons were not performed. Body weight changes and total feed intake were analyzed to determine the impact of the compounds on growth performance.

### 2.4. Sample Collection

At 24 h after diquat administration, all mice were euthanized under deep anesthesia in accordance with institutional animal welfare guidelines to minimize pain and distress. Serum was obtained from blood samples collected via the mouse orbital sinus using heparin-free capillaries, placed at room temperature for 30 min, and then centrifuged at 4 °C, 12,000 rpm for 10 min. After blood collection, all the animals were euthanized for cervical dislocation, and the thymus and spleen were carefully dissected and weighed. Organ indices were calculated as organ weight (mg) divided by body weight (g). A segment of the jejunum was excised and immediately immersed in 4% paraformaldehyde for histomorphology assessment. Further jejunum and liver samples were quickly collected, immediately frozen in liquid nitrogen, and stored at −80 °C for subsequent analysis of antioxidant parameters.

### 2.5. Serum Biochemical Analysis

Serum aspartate aminotransferase (AST; cat#C010-2-1) and alanine aminotransferase (ALT; cat#C009-2-1) were measured using commercial assay kits purchased from the Nanjing Jiancheng Bioengineering Institute (Nanjing, China). Serum D-lactate (D-LA) concentration was determined using a quantitative assay kit (Beyotime Biotechnology, cat# S0204S; Shanghai, China). Serum diamine oxidase (DAO) activity was measured according to the provided protocol using a commercial assay kit from the Nanjing Jiancheng Bioengineering Institute (cat# A088-3-1; Nanjing, China). All absorbance readings were obtained with a microplate reader (SpectraMax i3x, Molecular Devices, San Jose, CA, USA).

### 2.6. Antioxidant Capacity of the Liver and Jejunum

The total antioxidant capacity (TAOC; cat# S0116), superoxide dismutase (SOD) activity (cat# S0101S), glutathione peroxidase (GSH-Px) activity (cat# S0059S), and malondialdehyde (MDA) content (cat# S0131S) in liver and jejunal tissues were quantified using commercial kits from Beyotime Biotechnology Co., Ltd. (Shanghai, China). All experimental data were expressed relative to total protein content, measured using a BCA protein assay kit (cat# P0010; Beyotime, Shanghai, China) according to the manufacturer’s protocol. All absorbance readings were obtained with a microplate reader (SpectraMax i3x, Molecular Devices, San Jose, CA, USA).

### 2.7. Morphology Analysis

Jejunum samples were fixed in 4% paraformaldehyde, embedded in paraffin, sectioned at 5 μm, and stained with hematoxylin and eosin (H&E). Well-oriented villus–crypt units were selected for measurement. Villus height (VH; measured from villus–crypt junction to villus tip) and crypt depth (CD; measured from crypt base to villus–crypt junction) were quantified using a Leica DM750 microscope (Leica Microsystems GmbH, Wetzlar, Germany), and the villus height-to-crypt depth ratio (VCR) was calculated as villus height (μm) divided by crypt depth (μm).

### 2.8. Western Blotting

Liver and jejunal tissue proteins were isolated with RIPA buffer containing protease and phosphatase inhibitors. Following separation via SDS–PAGE, proteins were transferred to nitrocellulose membranes. After blocking, membranes were probed with specific primary antibodies and corresponding HRP-conjugated secondary antibodies. Detection was performed with enhanced chemiluminescence substrate (#34580; Thermo Fisher Scientific, Waltham, MA, USA), and band intensity was analyzed using ECL hyperfilm (AGFA, Mortsel, Belgium). The antibodies used were as follows: Nrf2 (#16396-1-AP), NQO1 (#11451-1-AP), MUC2 (#27675-1-AP) and Catalase (#21260-1-AP) from Proteintech (Wuhan, China); HO-1 (#AF5393), Occludin (#DF7504), and ZO-1 (#AF5154) from Affinity Biosciences Group Ltd. (Cincinnati, OH, USA). β-actin (#I102) served as the internal reference protein (Bioworld Biotech, Nanjing, China). The secondary antibody employed was anti-rabbit HRP (#7074) from Cell Signaling Technology (Shanghai, China).

### 2.9. Statistical Analysis

Statistical analysis was conducted with SPSS 26.0 using one-way ANOVA, followed by Duncan’s test for post-hoc comparisons. Before ANOVA, normality and homogeneity of variance were assessed via the Shapiro–Wilk and Levene’s tests, respectively. Data are expressed as mean ± standard error of the mean (SEM), with *p* < 0.05 defined as statistically significant and 0.05 ≤ *p* < 0.10 regarded as a trend. Figures were prepared using GraphPad Prism 10.

## 3. Results

### 3.1. Growth Performance

At the beginning of the experiment, no difference in initial BW was detected among the groups (*p* > 0.05) (Table 1). After the 27-day feeding period, no significant differences in BW or body weight gain (BWG) were observed between the T0 group and any treatment group (*p* > 0.05). However, 24 h after intraperitoneal diquat administration, mice in the T1 group exhibited a significant reduction in BW and BWG compared with the T0 group (*p* < 0.05). Compared with the T1 group, the T2 group did not markedly improve in BW (*p* > 0.05), whereas mice in the T4 group showed a significant increase (*p* < 0.05). The T3 and T5 groups displayed an increasing trend in BW (*p* < 0.10). Significantly, the body weights of mice in the T3, T4, and T5 groups did not differ from those in the T0 group (*p* > 0.05). Compared with the T0 group, the T2 group showed a 9.89% decrease in FI. In contrast, supplementation with EGCG derivatives improved FI, with the T4 group showing the most pronounced increase of 8.08%.

### 3.2. Immune Organ Index

Significant differences were observed in the thymus index and spleen index among the six groups (*p*< 0.01) (Table 2). Compared with the T0 group, the T1 group showed markedly reduced thymus and spleen indices (*p* < 0.01). Compared with the T1 group, the T2 group showed a significant increase in spleen index (*p* < 0.01) but no change in thymus index (*p* > 0.05), with both remaining lower than in the T0 group (*p* < 0.05). In contrast, the T3, T4, and T5 groups displayed significantly higher thymus and spleen indices than the T1 group (*p* < 0.01); the thymus index was restored to T0 levels (*p* > 0.05), whereas the spleen index, despite being elevated compared with the T1 group, remained lower than the T0 group.

### 3.3. Serum Biochemical Parameters

**Figure 1:**
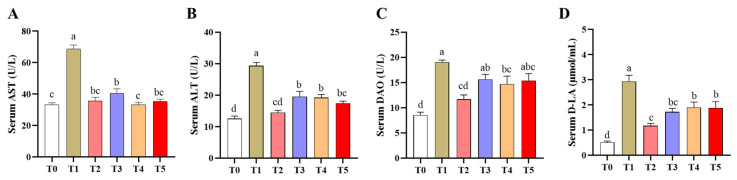
Effects of EGCG derivatives on serum biochemical parameters in mice. n = 6. (**A**) Serum aspartate aminotransferase (AST); (**B**) Serum alanine aminotransferase (ALT); (**C**) Serum diamine oxidase activity (DAO); (**D**) D-lactate contents (D-LA). T0, Control; T1, Diquat; T2, EGCG + Diquat; T3, Epigallocatechin octanoate + Diquat; T4, Epigallocatechin *p*-chloromethylbenzoate + Diquat; T5, Epigallocatechin ibuprofen ester + Diquat. ^a–d^ Different superscript letters indicate significant differences (*p* < 0.05).

Overall group comparisons revealed significant differences in serum AST, ALT, DAO, and D-LA levels among the six groups (*p* < 0.05) (Figure 1A–D). Serum AST, ALT, DAO, and D-LA levels were markedly elevated in the T1 group compared with the T0 group (*p* < 0.05) (Figure 1A–D). Compared with the T1 group, all treatment groups showed significant reductions in AST, ALT, and D-LA (*p* < 0.05), although these values remained above T0 levels (*p* < 0.05); only AST in the T4 group was fully restored to T0 levels (*p* > 0.05). In addition, serum DAO levels were significantly lower in the T2 and T4 groups than in T1 but did not return to T0 levels (*p* < 0.05).

### 3.4. Antioxidant Capacity of Jejunum and Liver

**Figure 2:**
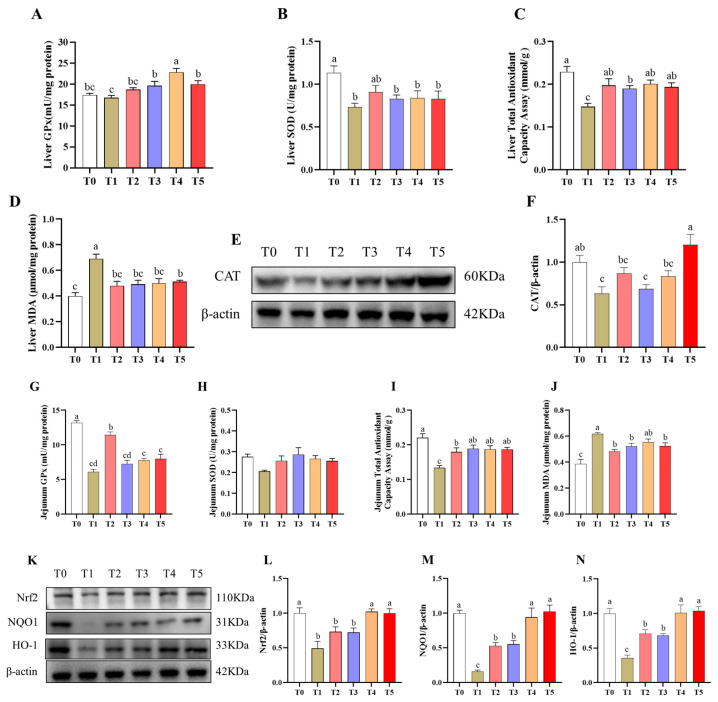
Effects of EGCG derivatives on the antioxidant capacity in the liver and jejunum of mice. n = 6. (**A**) Liver glutathione peroxidase (GSH-Px) activity; (**B**) Liver superoxide (SOD) activity; (**C**) Liver total antioxidant capacity (TAOC) activity; (**D**) Liver malondialdehyde (MDA) content; (**E**,**F**) The relative protein expression of catalase in the liver; (**G**) Jejunum glutathione peroxidase (GSH-Px) activity; (**H**) Jejunum superoxide (SOD) activity; (**I**) Jejunum total antioxidant capacity (TAOC) activity; (**J**) Jejunum malondialdehyde (MDA) content; (**K**–**N**) The relative protein expression of Nrf2, NQO1 and HO-1 in the jejunum. T0, Control; T1, Diquat; T2, EGCG + Diquat; T3, Epigallocatechin octanoate + Diquat; T4, Epigallocatechin *p*-chloromethylbenzoate + Diquat; T5, Epigallocatechin ibuprofen ester + Diquat. ^a–d^ Different superscript letters indicate significant differences (*p* < 0.05).

Significant differences were observed among the six groups in hepatic antioxidant parameters, including GPx, SOD, TAOC, and MDA levels, as well as CAT protein expression (*p* < 0.05) (Figure 2A–F). The T1 group mice exhibited marked oxidative stress, reflected by significantly reduced SOD and TAOC activities (*p* < 0.05) (Figure 2B,C), a decreasing trend in GPx activity (*p* < 0.10) (Figure 2A), and a pronounced elevation in MDA (*p* < 0.05) compared with the T0 group (Figure 2D). Compared with the T1 group, all treatment groups had significantly increased hepatic TAOC and GPx activities and lowered MDA levels (*p* < 0.05). Although the improvements in TAOC and MDA did not fully reach T0 values, GPx activity was restored to levels comparable with the T0 group (*p* < 0.05). Western blot analysis showed that the T1 group had markedly suppressed hepatic CAT protein expression compared with the T0 group (*p* < 0.05) (Figure 2E,F). Among all treatment groups, only the T5 group had significantly restored CAT abundance to a level comparable to the T0 group (*p* > 0.05).

Overall group comparisons revealed significant differences in jejunal antioxidant enzyme activities (GPx, TAOC, and MDA) and in Nrf2 pathway-related protein expression (Nrf2, HO-1, and NQO1) among the six groups (*p* < 0.05) (Figure 2G–N). The T1 group had similarly induced oxidative damage, as indicated by significantly lower GPx and TAOC activities and markedly higher MDA levels compared with the T0 group (*p* < 0.05) (Figure 2G,I,J). Compared with the T1 group, all treatment groups had significantly elevated TAOC activity and reduced MDA concentrations (*p* < 0.05), while only T2 had increased GPx activity (*p* < 0.05); however, antioxidant indices in all treatment groups remained lower than those of the T0 group. Western blot analysis showed that the T1 group had significantly decreased jejunal Nrf2, HO-1, and NQO1 protein expression compared with the T0 group (*p* < 0.05) (Figure 2K–N). Compared with the T1 group, the T2 and T3 groups had significantly increased NQO1 and HO-1 abundance (*p* < 0.05), although levels remained lower than those of the T0 group (*p* < 0.05). In contrast, the T4 and T5 groups had markedly increased protein expression of Nrf2, HO-1, and NQO1 (*p* < 0.05) and these markers fully restored to levels comparable to those of the T0 group (*p* > 0.05).

### 3.5. Jejunal Morphology and Barrier Function Analysis

**Figure 3:**
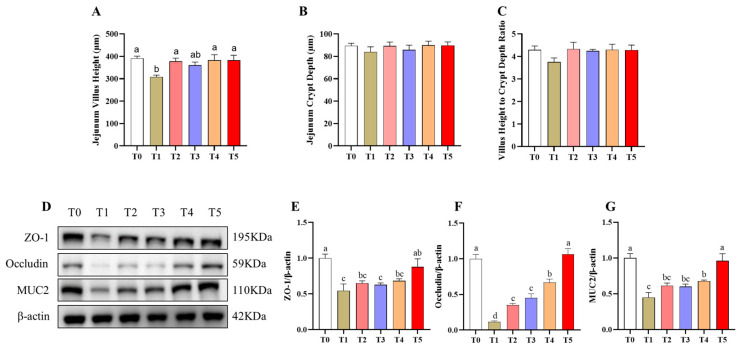
Effects of EGCG derivatives on jejunal morphology and barrier function in mice. n = 6. (**A**) Jejunal villus height; (**B**) Jejunal crypt depth; (**C**) Villus height to crypt depth ratio; (**D**–**G**) The relative protein expression of ZO-1, Occludin, and MUC2 in the jejunum. T0, Control; T1, Diquat; T2, EGCG + Diquat; T3, Epigallocatechin octanoate + Diquat; T4, Epigallocatechin *p*-chloromethylbenzoate + Diquat; T5, Epigallocatechin ibuprofen ester + Diquat. ^a–d^ Different superscript letters indicate significant differences (*p* < 0.05).

Significant differences were observed among the six groups in jejunal morphology (VH, CD, and VCR) and in intestinal barrier-related protein expression (ZO-1, Occludin, and MUC2) (*p* < 0.05) (Figure 3A–G). In jejunal morphology, the T1 group exhibited a significant reduction in VH compared with the T0 group (*p* < 0.05) (Figure 3A). All treatment groups had significantly increased VH compared with the T1 group (*p* < 0.05), restoring it to levels comparable to the T0 group (*p* > 0.05) (Figure 3B,C). Jejunal CD and VCR were not affected by any treatment (*p* > 0.05). For intestinal barrier function, the T1 group mice showed significant decreases in ZO-1, Occludin, and MUC2 protein abundance compared with the T0 group (*p* < 0.05) (Figure 3D–G). Compared with the T1 group, the T5 group had all three proteins significantly restored to T0-comparable levels (*p* < 0.05). In contrast, the other treatments elevated only Occludin expression (*p* < 0.05), which remained lower than T0 values.

## 4. Discussion

Previous studies have consistently confirmed that EGCG effectively alleviates oxidative stress induced by various stressors, including heat stress, oxidized lipids, and toxic substances [12,13,14,30]. However, the practical application of EGCG in animal production has been limited by its pronounced bitterness, which markedly reduces diet palatability and FI, thereby constraining its potential as a functional feed additive [19]. To address this limitation, three novel EGCG derivatives were chemically synthesized in the present study to reduce bitterness while preserving or enhancing antioxidant activity. By introducing hydrophobic groups, lipophilicity is adjusted, potentially improving cell membrane permeability and metabolic stability; at the same time, steric hindrance is used to reduce affinity for bitter taste receptors. Owing to the current limitations in synthesis yield, large-scale broiler trials were not feasible, and mice were therefore selected as an in vivo model for preliminary evaluation. A large number of studies have confirmed that diquat can lead to excessive ROS production, disrupt the antioxidant defense system, damage key metabolic organs such as the liver and small intestine, trigger acute inflammation, and ultimately inhibit growth performance [21,31,32,33]. Consistent with these reports, compared with the T0 group, BW and BWG in the T1 group were significantly reduced, confirming that oxidative damage severely inhibited growth and that the oxidative stress model had been successfully established. The addition of these three EGCG derivatives markedly attenuated the decline in growth performance caused by diquat. BW and BWG in the T4 group were significantly increased compared with the T1 group, while increasing trends were also observed in the T3 and T5 groups. Notably, BW in all three derivative-treated groups was restored to levels comparable with those of the T0 group, indicating a pronounced protective effect against oxidative stress-induced growth suppression. In contrast, the T2 group did not have significantly improved BW under oxidative stress conditions. Compared with the T0 group, the FI in the T2 group decreased by 9.86%, while the FI in both the T4 and T5 groups increased. These findings suggest that the bitterness and poor permeability of EGCG may have reduced its intake in vivo [19,34], thereby limiting its growth-promoting efficacy under oxidative stress. In contrast, the improved palatability of the EGCG derivatives likely enhanced their adequate intake, which, in turn, amplified their protective effects on growth performance. It should be acknowledged that while FI was used in this study as an indicator of palatability, it is influenced by multiple factors, including the animals’ overall health and metabolic status. Therefore, the observed improvements in FI in the T4 and T5 groups, although consistent with our design objective of reducing bitterness, do not constitute direct proof of improved palatability. Future studies should incorporate more direct assessments of bitterness and palatability to definitively establish whether the chemical modifications successfully reduce the bitter taste of EGCG. Furthermore, despite efforts to control the impact of individual differences on the results through random grouping, weight balancing, standardized feeding conditions, and other measures during the experimental design, the sample size of 6 mice per group remains relatively small for growth performance indicators such as BW and FI. In future broiler trials, a larger sample size will be necessary to validate these preliminary observations and to better account for the inherent variability in growth traits.

The thymus and spleen are central immune organs that play essential roles in maintaining immune homeostasis [35]. Evidence indicates that oxidative stress triggers apoptosis and suppresses proliferation in immune cells, resulting in structural atrophy of these organs and consequent immune dysfunction [36,37]. In the present study, diquat administration markedly decreased both thymus and spleen indices, indicating that acute oxidative stress severely impaired immune organ development and function. This finding is consistent with previous reports showing that diquat-induced oxidative injury causes pronounced immunosuppression in mice and poultry [38,39]. Compared with the T1 group, EGCG supplementation significantly increased the spleen index. However, it did not improve the thymus index, and both indices remained markedly lower than those of the T0 group. These results suggest that the immune-protective effect of EGCG under acute oxidative stress conditions was limited. In contrast, all three EGCG derivatives (EGCO, EGCP, and EGCI) exerted substantially stronger protective effects on immune organ development. The thymus indices in these groups were significantly elevated compared with those in the T1 group and fully restored to levels comparable to those in the T0 group. Meanwhile, the spleen indices were also significantly increased, although they remained lower than those of the T0 group. Previous studies have demonstrated that enhancing antioxidant capacity effectively attenuates oxidative stress-induced immune organ atrophy and restores immune function [40], findings that are in good agreement with the present findings.

ALT and AST are classical biochemical markers of hepatocellular injury. Prior research has shown that diquat-induced oxidative stress results in substantial hepatocellular damage, accompanied by a pronounced elevation in serum ALT and AST levels [21,23,24], findings consistent with the present findings. Additionally, key hepatic antioxidant enzymes, including SOD, TAOC, and GPx, exhibited significantly reduced activity. In contrast, MDA levels were markedly increased following diquat challenge, indicating a pronounced impairment of hepatic antioxidant defense capacity and an aggravation of lipid peroxidation. These alterations are characteristic features of classical oxidative stress injury models [8,21,40]. After supplementation with EGCG derivatives, especially EGCI, serum ALT and AST levels were significantly reduced. Meanwhile, hepatic TAOC and GPx activities were markedly enhanced, and MDA levels were significantly decreased, suggesting that both hepatic functional injury and oxidative damage were effectively ameliorated. Similar protective trends were also observed in the jejunum, indicating that EGCG derivatives exerted a systemic protective effect against diquat-induced oxidative stress rather than an organ-specific action.

Serum DAO and D-LA are widely recognized as sensitive biochemical markers of intestinal mucosal integrity and permeability [41]. In contrast, jejunal morphology and the expression of tight junction proteins (ZO-1, Occludin, and Claudin-1) reflect the structural and molecular integrity of the intestinal barrier [42]. In the present study, intraperitoneal injection of diquat significantly elevated serum DAO and D-LA levels, accompanied by marked impairment of jejunal villus architecture and significant downregulation of ZO-1, Occludin, and MUC2 protein expression, further confirming the destructive effects of oxidative stress on intestinal morphology and barrier function. Diquat induces excessive generation of reactive oxygen species, which directly damages intestinal epithelial cells and disrupts tight junctions, leading to loosening of intercellular connections and increased intestinal permeability, thereby facilitating the abnormal translocation of DAO and D-LA into the peripheral circulation [39,43]. Following supplementation with EGCG derivatives, especially EGCI, serum DAO and D-LA levels were significantly reduced. In contrast, jejunal villus height and the protein expression levels of ZO-1, Occludin, and MUC2 were markedly upregulated, indicating that both intestinal morphology and barrier function were effectively restored. These findings suggest that EGCG derivatives preserve epithelial integrity by maintaining tight junction protein expression and stabilizing intercellular junctional structures, thereby reducing intestinal permeability and limiting the abnormal translocation of bacterial metabolites into the bloodstream [11,26]. By contrast, the protective effects observed in the T2 group were relatively modest, which may be attributed to reduced feed intake due to its pronounced bitterness, resulting in insufficient systemic bioavailability and, consequently, limiting its intestinal protective efficacy.

The Nrf2/HO-1 signaling pathway is a pivotal redox-sensitive defense mechanism that regulates the transcription of a series of antioxidant and cytoprotective genes to maintain cellular redox homeostasis [44,45]. It has been reported that EGCG activates the Nrf2/HO-1 pathway and subsequently upregulates the expression of antioxidant-related genes, such as CAT, GPx, and HO-1 [29,30], thereby enhancing reactive oxygen species scavenging and suppressing lipid peroxidation. In the present study, Western blot analysis further demonstrated that diquat significantly inhibited hepatic CAT protein expression and concomitantly downregulated Nrf2, HO-1, and NQO1 protein levels in the jejunum. In contrast, EGCI supplementation markedly restored protein expression to levels comparable to those of the T0 group. These findings preliminarily suggested that EGCG derivatives may strengthen hepatic and intestinal antioxidant capacity by activating the Nrf2/HO-1 pathway, thereby conferring systemic protection against oxidative stress. Further studies employing genetic knockout or knockdown approaches are needed to validate the causal relationship.

It is noteworthy that all compounds were administered at a fixed dose of 300 mg/kg, resulting in different molar doses due to their varying molecular weights. The molecular weights of EGCG, EGCO, EGCP, and EGCI were 458.4 g/mol, 432.5 g/mol, 458.9 g/mol, and 494.5 g/mol, respectively, corresponding to molar doses of 654.5 μmol/kg, 693.6 μmol/kg, 653.7 μmol/kg, and 606.7 μmol/kg. Importantly, although the molar dose of EGCI was lower than that of EGCG, EGCI exhibited superior protective effects, suggesting that the enhanced efficacy of EGCI is attributed to its chemical modifications rather than simply to a higher molar dose of the EGC core. In contrast, EGCP, with a molar dose comparable to that of EGCG, showed similar efficacy, further supporting that the observed advantage of EGCI arises from its unique structural features.

However, the current experimental design does not allow us to fully clarify whether the significant effect of EGCI is due to the enhanced EGC-mediated antioxidant activity through the Nrf2 pathway or the intrinsic anti-inflammatory effect of its ibuprofen part. It must be recognized that EGCI is a new chemical substance, and its metabolic pathway in vivo, whether it exists in the form of a complete compound or releases free ibuprofen, remains to be further elucidated. Therefore, comprehensive pharmacokinetic and metabolic studies must be conducted in future broiler trials to determine the distribution of EGCI and its metabolites, as well as residue depletion studies to determine the appropriate withdrawal period, and submit complete documentation to obtain regulatory approval for its use as a novel feed additive.

Beyond the biological efficacy demonstrated in this study, the translational potential of EGCG derivatives, particularly EGCI, as commercial feed additives depends critically on their economic feasibility. While a comprehensive cost–benefit analysis would require large-scale production, a preliminary assessment based on available literature and current data suggests promising prospects. The synthesis of EGCI involves conventional organic chemistry methods using readily available reagents and starting materials, suggesting that its scaled-up production costs could be comparable to those of existing commercial feed additives. Studies have shown that oxidative stress can reduce broiler growth performance [46,47]; thus, even partial mitigation of these losses could yield significant economic returns. These preliminary estimates support the potential of EGCI as an economically viable feed additive. However, definitive conclusions await future investigations involving industrial-scale synthesis and broiler feeding trials to directly evaluate both production benefits and cost-effectiveness.

Collectively, the present study demonstrated that the three novel EGCG derivatives, especially EGCI, confer significant systemic antioxidant protection in a diquat-induced murine model of acute oxidative stress. While physiological and metabolic differences exist between mammals and poultry, the core oxidative stress response pathway (e.g., the Nrf2 signaling axis) is highly conserved across species. Therefore, the mechanistic insights gleaned from this mouse model provide valuable translational clues for applications in poultry. Nevertheless, this work constitutes a pivotal first step in lead compound screening, primarily focused on validating the in vivo bioactivity and elucidating the underlying principles. Future research will focus on optimizing the synthetic route to improve yield, followed by direct evaluation in broiler feeding trials. These essential next steps will assess the practical impacts on growth performance, health metrics, and economic viability, thereby bridging the gap from mechanistic proof-of-concept to applied development.

## 5. Conclusions

In conclusion, the chemically synthesized EGCG derivatives, especially EGCI, markedly attenuated diquat-induced growth suppression and oxidative injury in the liver and intestine of mice. These protective effects were mainly attributed to improvements in jejunal morphology and barrier integrity, reduced intestinal permeability, and coordinated enhancement of antioxidant capacity in both the liver and the intestine. Among the derivatives tested, EGCI exhibited the most favorable overall profile. Compared with EGCG, the EGCI exhibited reduced bitterness and improved palatability, which was beneficial for maintaining normal feed intake. Mechanistically, EGCI exerted its antioxidant effects, at least in part, by activating of the Nrf2/HO-1 signaling pathway and upregulating antioxidant-related proteins. Collectively, these results highlight EGCI as a lead candidate worthy of further investigation. These findings provide a solid theoretical basis for the future application of EGCG derivatives, particularly EGCI, in broiler production.

## Figures and Tables

**Table 1:** Effects of EGCG derivatives on growth performance in mice.

Item ^1^	1 Day BW, g	27 Day BW, g	28 Day BW, g	1–27 Days BWG, g	27–28 Day BWG, g	FI ^2^, g
T0	29.77	41.22	41.35 ^ab^	11.45	0.13 ^a^	149.52
T1	30.23	41.68	39.70 ^b^	11.45	−1.98 ^b^	142.43
T2	30.13	40.67	39.38 ^b^	10.53	−1.28 ^b^	134.73
T3	29.22	42.37	40.25 ^ab^	13.15	−2.12 ^b^	143.56
T4	29.25	43.45	41.92 ^a^	14.20	−1.53 ^b^	161.60
T5	29.40	41.48	40.52 ^ab^	12.08	−0.97 ^ab^	151.06
SEM	0.32	0.30	0.27	0.45	0.19	-
*p*-value	0.916	0.102	0.042	0.201	0.002	-

^1^ T0, Control; T1, Diquat; T2, EGCG + Diquat; T3, Epigallocatechin octanoate + Diquat; T4, Epigallocatechin *p*-chloromethylbenzoate + Diquat; T5, Epigallocatechin ibuprofen ester + Diquat. n = 6. ^2^ Feed intake was the average feed intake per mouse during the 28-day trial period in each group. Abbreviations: BW, body weight; BWG, body weight gain; FI, feed intake. ^a,b^ Different superscript letters indicate significant differences (*p* < 0.05).

**Table 2:** Effects of EGCG derivatives on immune organ index in mice.

Item ^1^	Thymus, mg	Spleen, mg	Thymus Index ^2^, mg/g	Spleen Index ^3^, mg/g
T0	97.75 ^a^	152.48 ^a^	23.54 ^a^	36.87 ^a^
T1	60.97 ^b^	87.87 ^c^	15.30 ^b^	22.16 ^c^
T2	60.17 ^b^	113.20 ^bc^	15.25 ^b^	28.40 ^b^
T3	93.33 ^a^	121.42 ^b^	22.13 ^a^	28.69 ^b^
T4	89.60 ^a^	133.08 ^ab^	21.79 ^a^	32.33 ^ab^
T5	86.10 ^a^	116.82 ^b^	21.16 ^a^	28.7 ^b^
SEM	2.97	4.54	0.64	0.99
*p*-value	<0.001	<0.001	<0.001	<0.001

^1^ T0, Control; T1, Diquat; T2, EGCG + Diquat; T3, Epigallocatechin octanoate + Diquat; T4, Epigallocatechin *p*-chloromethylbenzoate + Diquat; T5, Epigallocatechin ibuprofen ester + Diquat. n = 6. ^2^ Thymus index was calculated as thymus weight (mg) divided by body weight (g). ^3^ Spleen index was calculated as spleen weight (mg) divided by body weight (g). ^a–c^ Different superscript letters indicate significant differences (*p* < 0.05).

## Data Availability

The data presented in this study are available on request from the corresponding author.
